# The complete chloroplast genome of *Strobilanthes crispus* (Acanthaceae)

**DOI:** 10.1080/23802359.2022.2111979

**Published:** 2022-08-26

**Authors:** Guoqing Wan, Yanhui Liu, Yanjun Zheng, Xuefeng Gu, Changlian Lu

**Affiliations:** a Shanghai Key Laboratory of Molecular Imaging, Zhoupu Hospital, Shanghai University of Medicine and Health Sciences, Shanghai, PR China; b School of Pharmacy, Shanghai University of Medicine and Health Sciences, Shanghai, PR China

**Keywords:** *Strobilanthes crispus*, complete chloroplast genome, phylogenetic analysis

## Abstract

We reported and characterized the complete chloroplast genome sequence of *Strobilanthes crispus* Blume 1826. *Strobilanthes crispus* belongs to the Acanthaceae family and has a number of local names including Batuzin, Bayam Karang, Kotz Bellin, and Pekka Batu, which is native to Malaysian with diverse beneficial uses. Green leaves were determined using next-generation sequencing. We found that the entire chloroplast genome of *S. crispus* was 144,987 bp in length, included four segments, named a large single-copy (LSC) region (92,556 bp), a small single-copy (SSC) region (17,783 bp), and a pair inverted repeat regions (IRs) (17,324 bp in each), respectively. The chloroplast genome of *S. crispus* contained a total of 129 functional genes, including 84 protein-coding genes, 37 transfer RNAs (tRNAs), and eight ribosomal RNA (rRNA) genes. The phylogenetic tree reconstructed by nine chloroplast genomes reveals that *S. crispus* is most closely related to *Strobilanthes bantonensis* and *Strobilanthes cusia*.

*Strobilanthes crispus*, known as the black faced general, is native to Malaysia. In folk medicine, *S. crispus* has been used to treat some diseases including diuretic, anticancer, antidiabetic, antilytic, and laxative for decades (Sunarto 1977; Goh 2004). It belongs to a family of *Acanthopanax senticosus* and has many local names, including Batuzin, Bayam Karang, Kotz Bellin, and Pekka Batu. In addition, in previous studies, its phylogenetic position is unclear because of the lack of genomic information. Here, we characterized the complete chloroplast genome sequence of *S. crispus* based on the genome skimming sequencing data. These genomic data can provide intragenic information to clarify the taxonomic characteristics and valuable information about the evolution of *Strobilanthes*.

The samples of *S. crispus* were collected from Zhao'an County, Zhangzhou, Fujian Province, China (23°71N, 117°18E). The voucher specimen was deposited in Shanghai University of Medicine and Health Sciences under the voucher number SUMHS020190707 (Guoqing Wan, wangq@sumhs.edu.cn). The total genomic DNA was extracted from the fresh leaves of *S. crispus* using the DNeasy Plant Mini Kit (Qiagen, Valencia, CA). The DNA was stored at 80 °C in our lab. The whole-genome sequencing was conducted on the BGISEQ-500 sequencing platform from Hefei Biodata Biotechnologies Inc. (Hefei, China). The complete chloroplast genome was assembled using the program SPAdes assembler 3.10.0 using default settings (Bankevich et al. 2012). Software GeSeq (Tillich et al. 2017) was used to annotate the chloroplast genes. All transfer RNA (tRNA) genes were further verified by using tRNAscan-SE 2.0 program (Chan et al. 2021).

The chloroplast genome sequence of *S. crispus* was submitted to NCBI (GenBank accession no. MW411449), consisting of two inverted repeat regions (IRs) of 17,324 bp, a large single-copy (LSC) with 92,556 bp, and a small single-copy (SSC) region with 17,783 bp. The content of overall GC in the complete chloroplast genome of the *S. crispus* is 38.2%, and the corresponding values in LSC, SSC, and IR regions are 36.5%, 32.6%, and 45.6%, respectively. The chloroplast genome of *S. crispus* is predicted to contain 129 genes with different functional classifications, including 84 protein-coding genes, 37 tRNA genes, and eight ribosomal RNA (rRNA) genes. Among these genes, five protein-coding, six tRNA, and four rRNA genes were duplicated in the IR regions.

To investigate its taxonomic status, alignment was performed on the nine complete chloroplast genome sequences using MAFFT v7.307 (Katoh and Standley 2013), and a maximum-likelihood (ML) tree was constructed with 1000 bootstrap replicates in FastTree v2.1.10 (Price et al. 2010). *Dicliptera mucronata* (MK848596) and *Justicia flava* (NC_044862) were used as outgroups to construct the phylogenetic tree. The ML phylogenetic tree shows that *S. crispus* is most closely related to *Strobilanthes bantonensis* (MT576695) and *Strobilanthes cusia* (NC_037485) in Acanthaceae (Figure 1).

**Figure 1.**
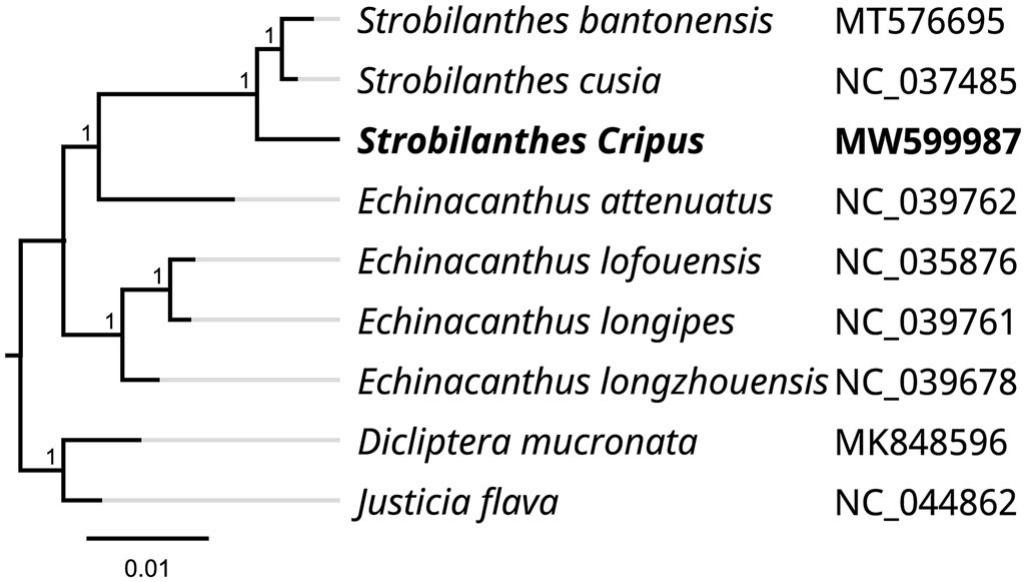
Phylogenetic tree inferred by maximum-likelihood (ML) method based on the complete chloroplast genome of nine representative species. A total of 1000 bootstrap replicates were computed and the bootstrap support values were shown at the branches. Accession numbers are shown in the figure.

## Data Availability

The genome sequence data that support the findings of this study are openly available in GenBank of NCBI (https://www.ncbi.nlm.nih.gov/nuccore/MW599987/) under the accession no. MW599987. The associated BioProject, SRA, and Bio-Sample numbers are PRJNA701834, SRR13720062, and SAMN17914799, respectively.
